# Supplementary material for: Evaluation of In Vitro Distribution and Plasma Protein Binding of Selected Antiviral Drugs (Favipiravir, Molnupiravir and Imatinib) against SARS-CoV-2
Source: Int J Mol Sci. 2023 Feb 2;24(3):2849. doi: 10.3390/ijms24032849 (PMC9917862; doi:10.3390/ijms24032849)
Supplement: Supplementary file 1 [file ijms-24-02849-s001.zip › ijms-2118605-supplementary.pdf]

SUPPLEMENTARY INFORMATION

Evaluation of in vitro distribution and plasma protein binding of selected antiviral drugs (favipiravir, molnupiravir and imatinib) against SARS-CoV-2

Orsolya Dömötör*, Éva A. Enyedy

FIGURES

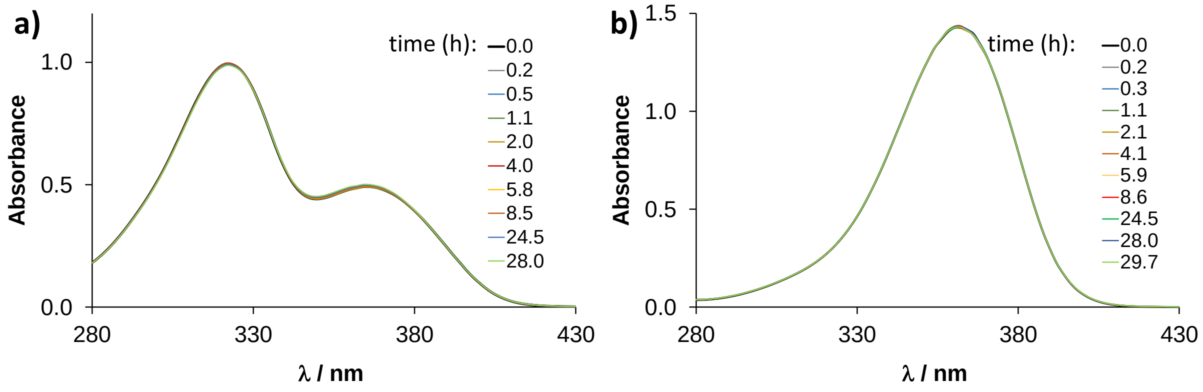

Figure S1. UV-vis absorption spectra of FAVI at pH 2.0 (a) and pH 7.4 (b) followed in time as it is indicated in the figure. {c_{FAVI} = 156 μM; t = 25°C}.

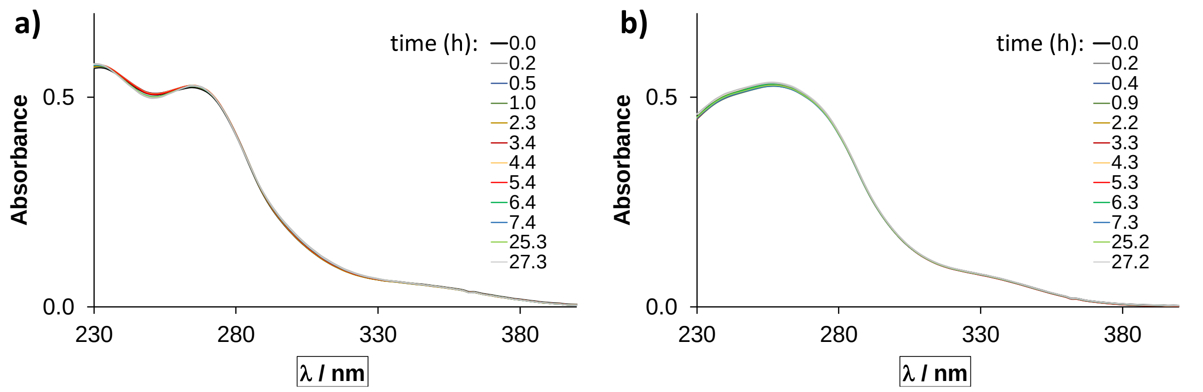

Figure S2. UV-vis absorption spectra of IMA at pH 2.0 (a) and pH 7.4 (b) followed in time as it is indicated in the figure. {c_{IMA} = 16 μM; t = 25°C}.

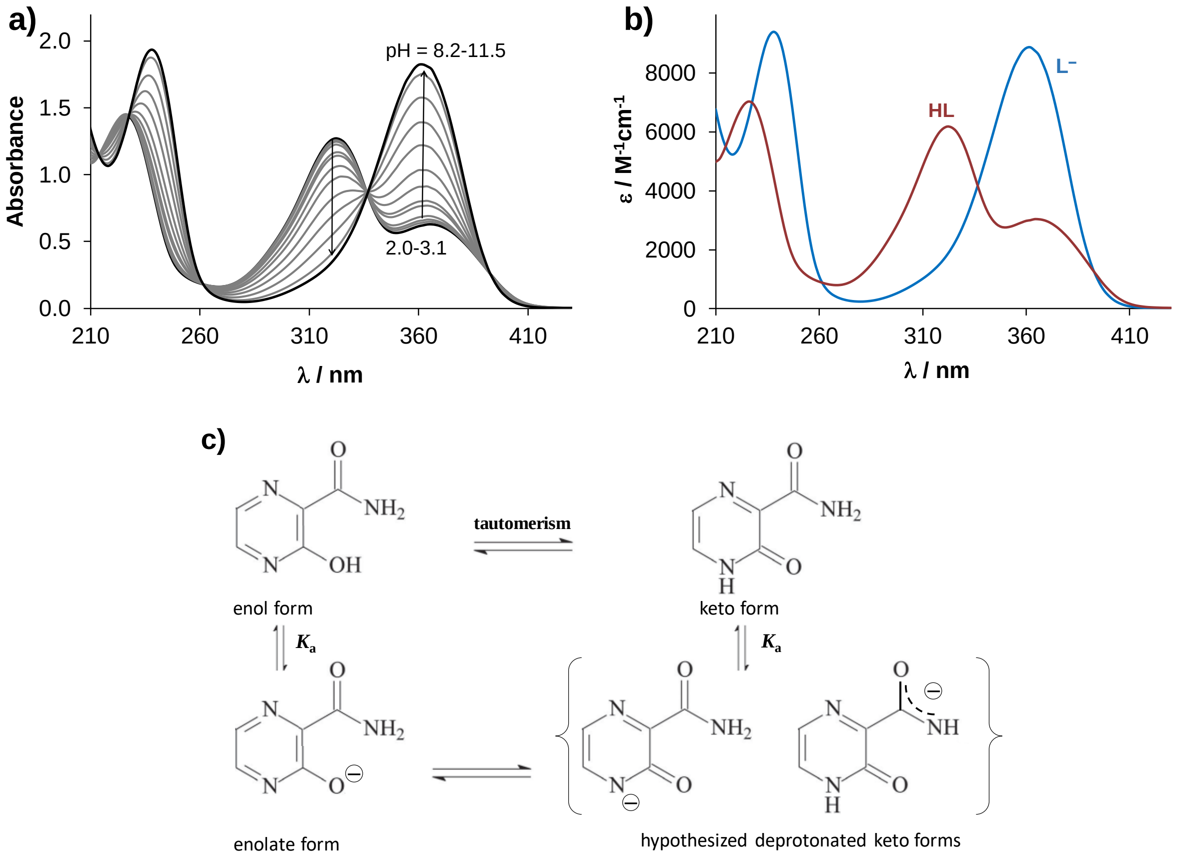

Figure S3. (a) Absorption spectra of FAVI recorded in the pH 2.0 – 11.5; (b) computed individual molar spectra of the two forms HL and L^- and (c) tautomerism and presumed deprotonation equilibria for the enol and keto forms of FAVI {c_{FAVI} = 206 μM; I = 0.1 M (KCl), t = 25 °C}.

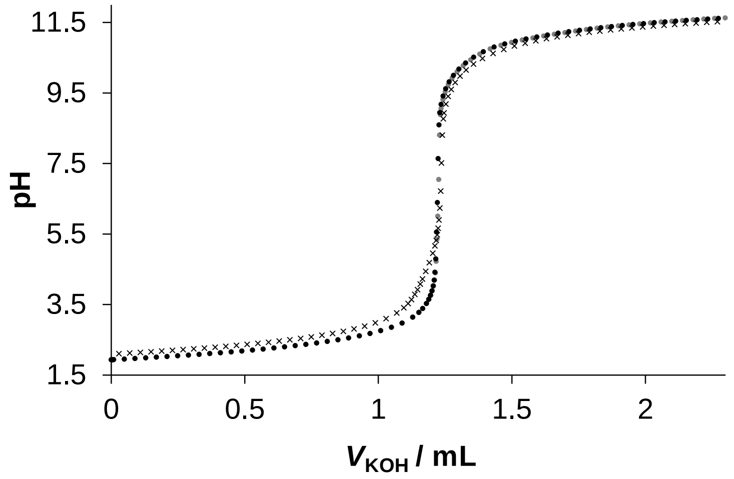

Figure S4. pH-potentiometric titration curve of MOLNU prepared freshly (●), stored in stock solution for 1 day (●) and back-acidification of the titrated (freshly prepared) sample (×). {c_{MOLNU} = 1.0 mM; I = 0.1 M (KCl), t = 25 °C}.

Note: grey dots overlap almost perfectly with the black ones, they can be distinguished only in the basic pH-range.

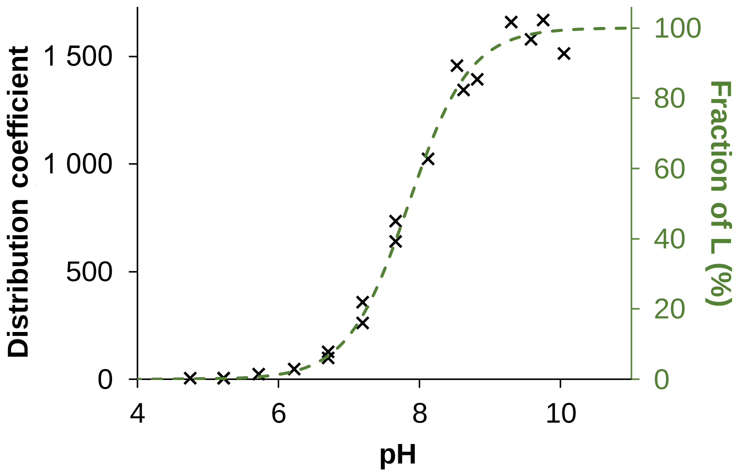

Figure S5. Distribution coefficients (*D*) of IMA determined by *n*-octanol/water partitioning at various pH values plotted together with the calculated molar fraction of the neutral L form {I = 0.1 M (KCl); t = 25 °C}.

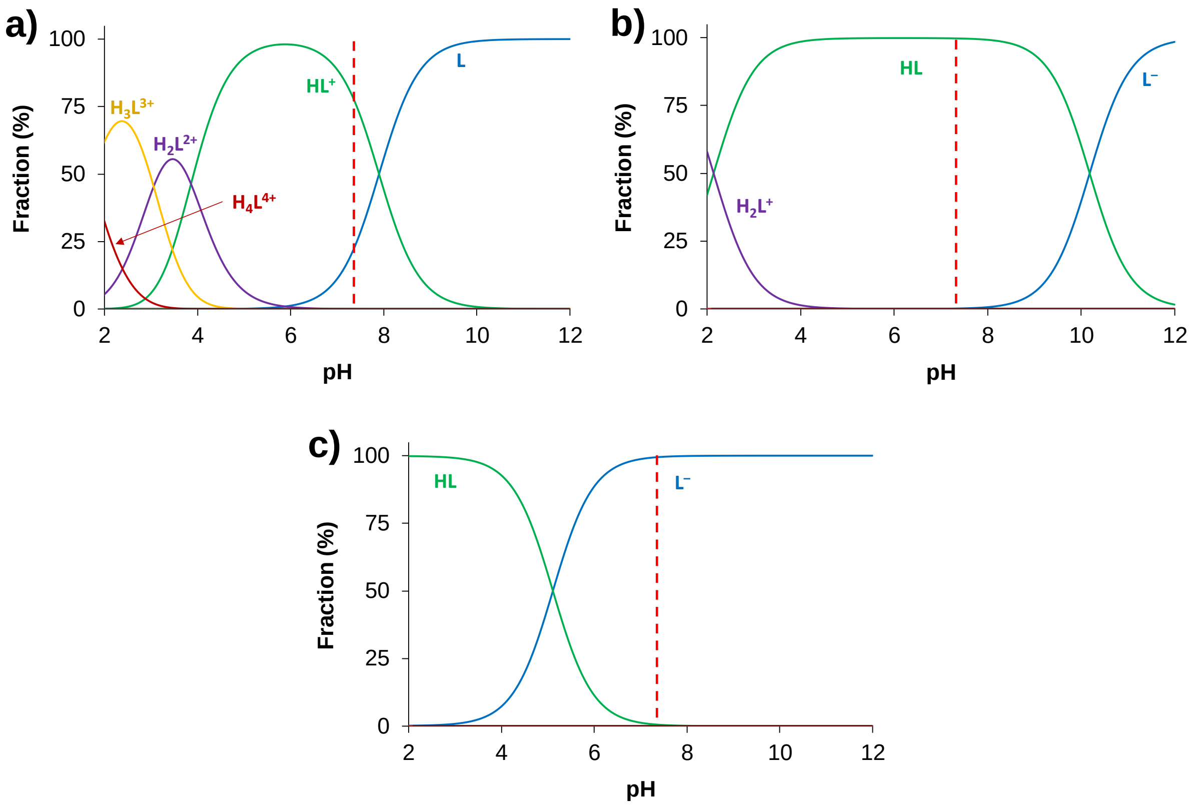

Figure S6. Concentration distribution curves of (a) IMA, (b) MOLNU and (c) FAVI, the dashed red line indicates the distribution at pH 7.4 {I = 0.1 M (KCl); t = 25 °C}

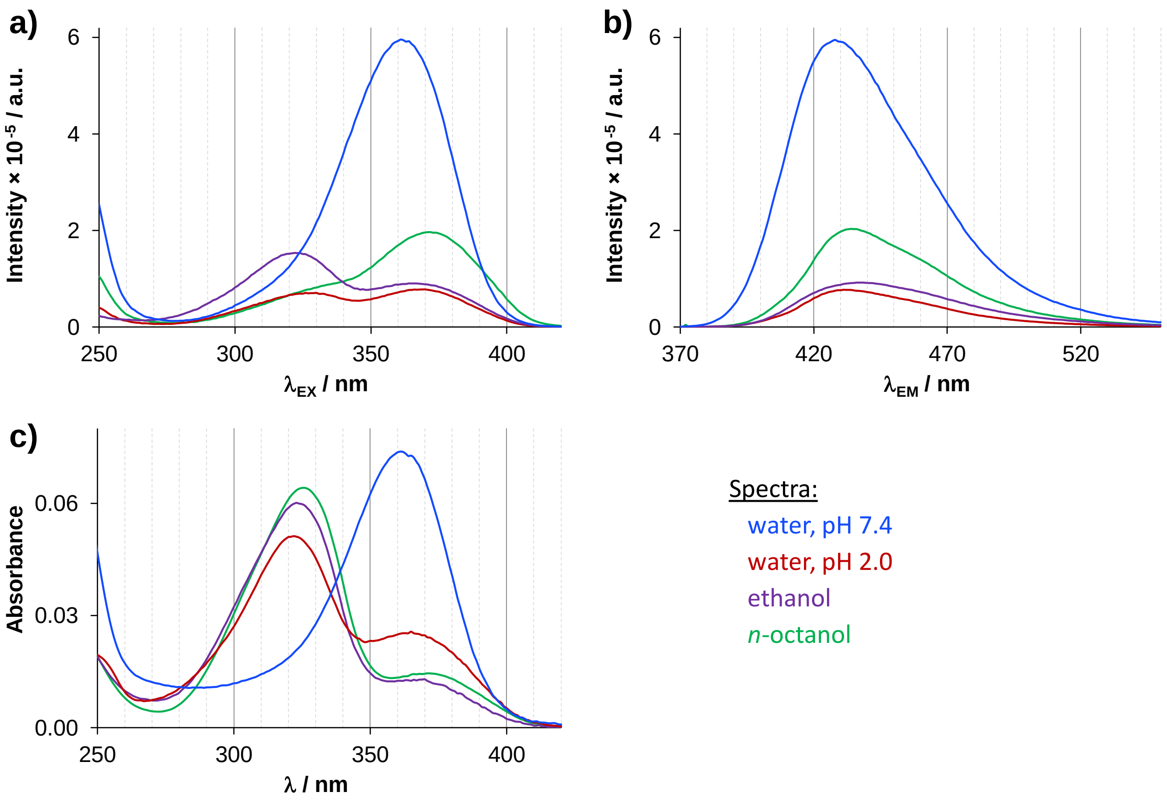

Figure S7. Fluorescence (a) excitation (λ_{EM} = 430 nm), (b) emission (λ_{EX} = 360 nm) and (c) UV-vis absorption spectra of FAVI recorded in various solvents {c_{FAVI} = 10 μM; t = 25°C}.

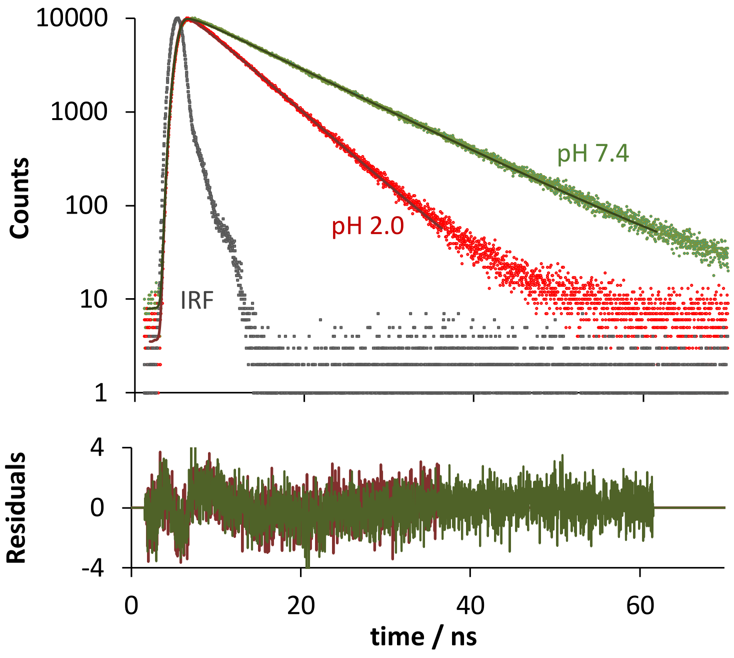

Figure S8. Fluorescence intensity decay of FAVI at pH = 2.0 and 7.4 together with the fitted curves $\tau_{pH\ 2.0}$ = 5.64 ns and $\tau_{pH\ 7.4}$ = 10.00 ns, respectively; IRF: instrument response function recorded for Ludox®; bottom: residual plot for the fitting of each decay {c_{FAVI} = 10 µM; λ_{EX} = 360 nm; λ_{EM} = 430 nm; pH = 7.4 (PBS); t = 25 °C}.

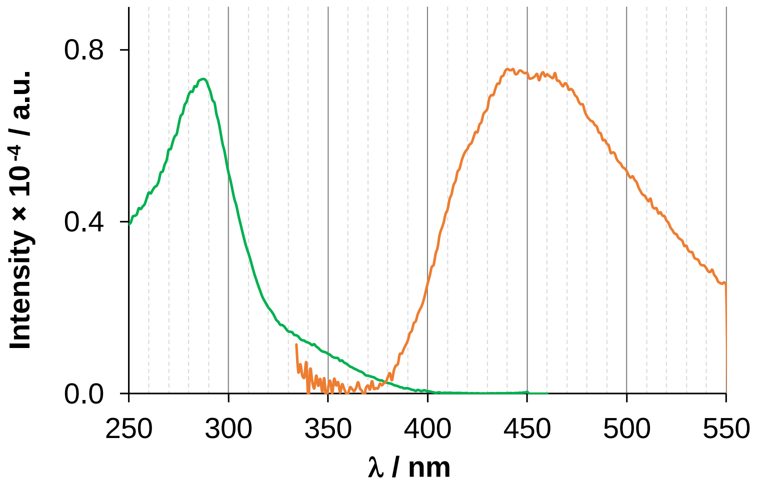

Figure S9. Fluorescence excitation (green) and emission (orange) spectra of IMA in *n*-hexane {c_{IMA} = 10 µM; t = 25°C}.

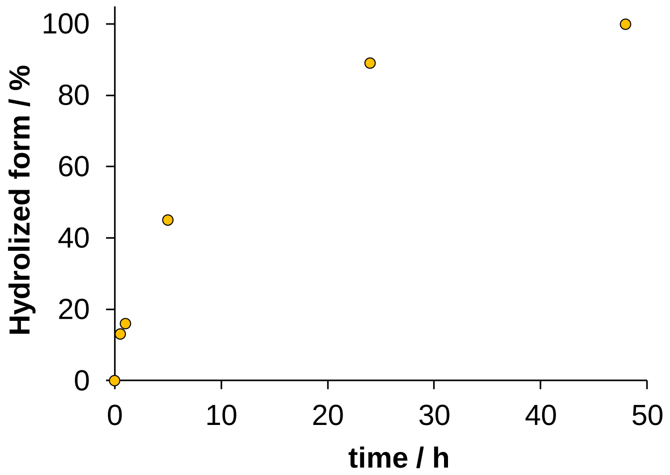

Figure S10. Calculated percentages of h-MOLNU appearing in the MOLNU – blood serum system. Calculations are based on the baseline corrected ^{1}H NMR peak integrals recorded at *ca*. δ = 1.2 ppm {c_{MOLNU} = 1 mM; blood serum: 2-fold diluted; pH = 7.4 (PBS), 10% (v/v) D_2O/H_2O; t = 25 °C}.

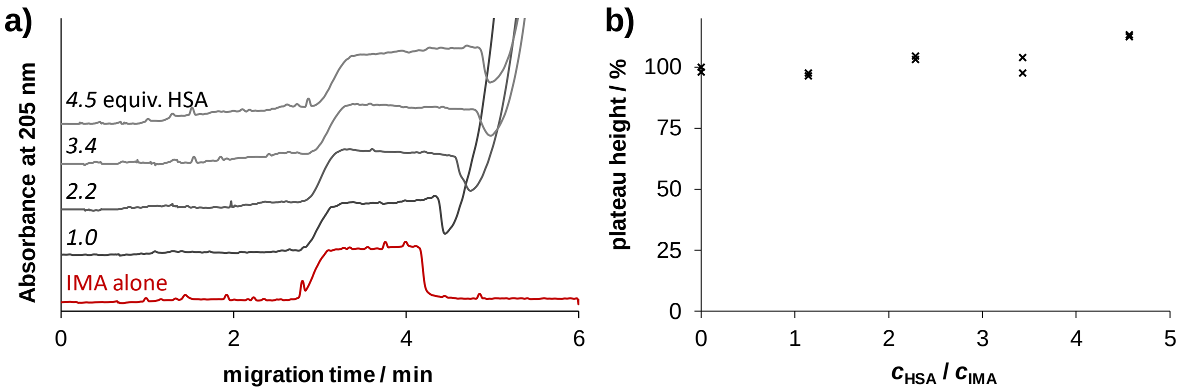

Figure S11. (a) Electropherograms recorded by FACE for the HSA – IMA system at constant concentration of IMA and (b) plateau heights collected for free IMA {c_{IMA} = 15 µM; c_{HSA} = 0–67.5 µM; BGE: pH = 7.4 (PBS); voltage: 10 kV, current: 180 µA; t = 25 °C}.

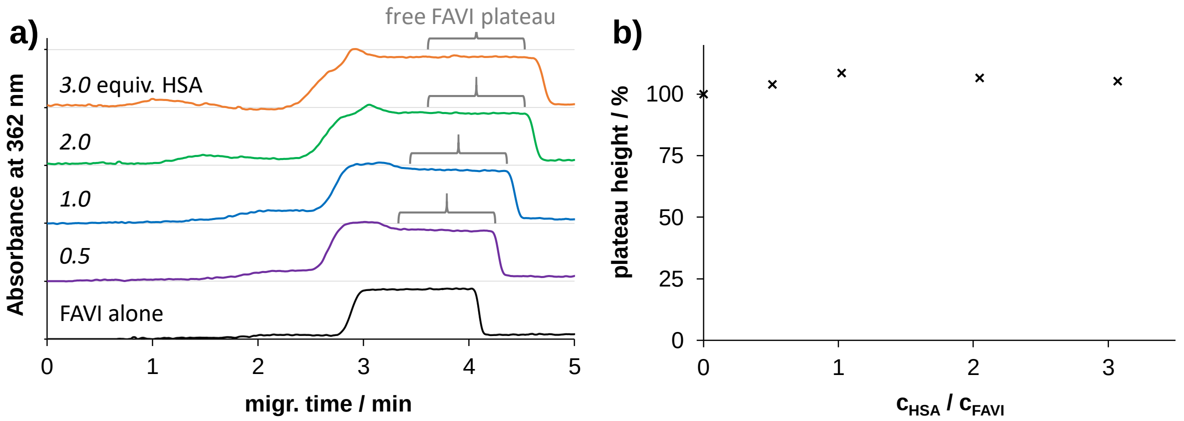

Figure S12. Electropherograms recorded by FACE for the HSA – FAVI system at constant concentration of FAVI titrated by HSA (a); and plateau heights collected for free FAVI (b) {c_{FAVI} = 29 µM; c_{HSA} = 0–87 µM; BGE: pH = 7.4 (PBS); voltage: 10 kV, current: 180 µA; t = 25 °C}.

Note: the free fraction of FAVI, as negatively charged small molecule, migrates after HSA, which later has negligible absorption at 362 nm.

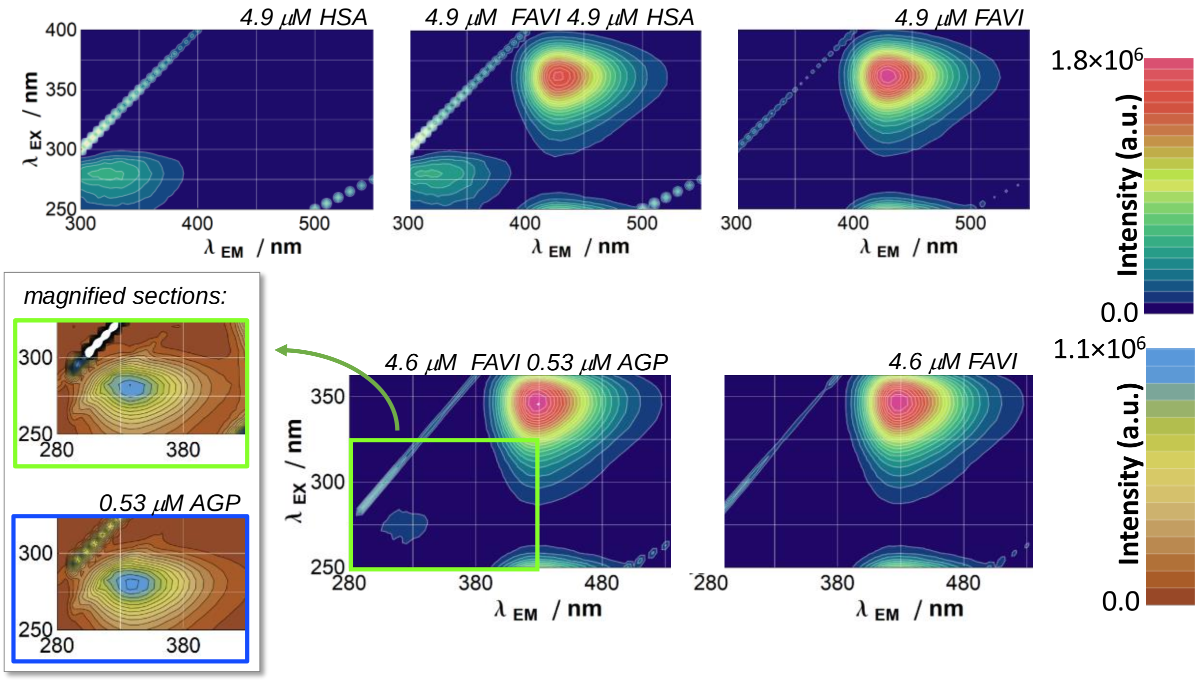

Figure S13. Three-dimensional fluorescence spectra of the HSA–FAVI and AGP–FAVI systems {pH = 7.4 (PBS); t = 25°C}. *Note: spectra for the AGP–FAVI system were recorded at 1.5 nm/1.5 nm slit widths (bluish colored spectra) and at 5 nm/5 nm slit widths (small brownish colored spectra) as well, that is the reason for the use of two different intensity scales; multiplying factor is about 10-fold between the two scales.*

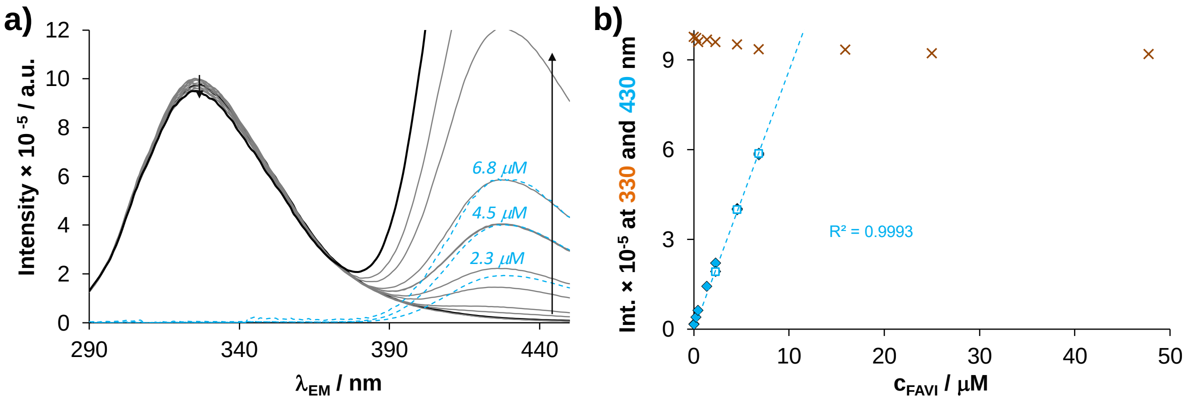

Figure S14. (a) Quenching of AGP fluorescence in the presence of FAVI and emission spectra of FAVI alone (cyan dashed spectra, see concentrations of FAVI in the figure; (b) recorded emission intensities at 330 nm (×) and 430 nm (♦) together with the fluorescence of FAVI alone (□) {c_{AGP} = 0.58 μM; c_{FAVI} = 0–48 μM; λ_{EX} = 280 nm; pH = 7.4 (PBS); t = 25 °C}.

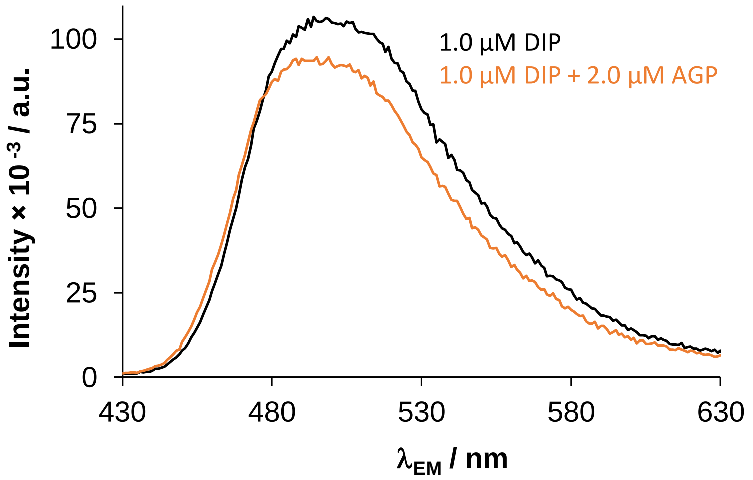

Figure S15. Fluorescence emission spectra of DIP in the absence and presence of 2 equiv. AGP {λ_{EX} = 400 nm; pH = 7.4 (PBS), t = 25 °C}.

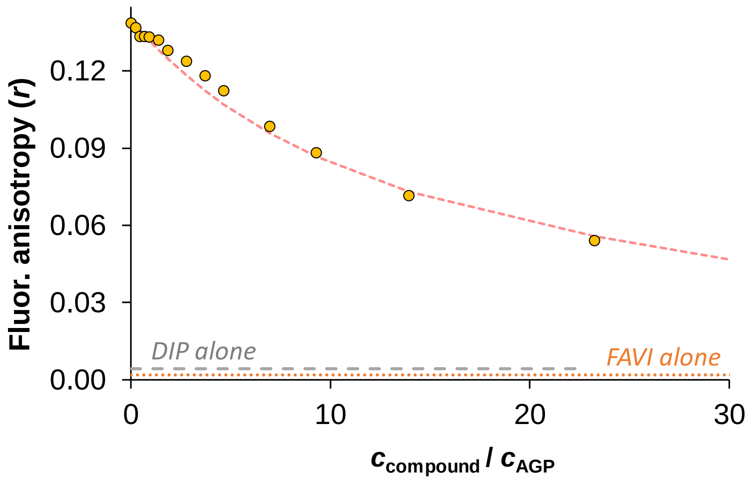

Figure S16. Reduction of the fluorescence anisotropy (r) of the DIP–AGP system by the addition of FAVI (●). Anisotropy signal of free DIP and FAVI is indicated with grey dashed and orange dotted line, respectively. Red dashed line was calculated for the simple physical mixtures containing [DIP–AGP], DIP and FAVI by the use of individual anisotropy values ($r_{[DIP\text{-}AGP]}$ = 0.25, r_{DIP} ≈ 0.003, r_{FAVI} ≈ 0.002) and individual contributions to the overall fluorescence intensity ($Int.(\mathrm{rel})_{[DIP\text{-}AGP]} \approx Int.(\mathrm{rel})_{DIP}$ = 1.0, $Int.(\mathrm{rel})_{FAVI}$ = 0.016) {c_{AGP} = 2.0 µM; c_{DIP} = 1.0 µM; λ_{EX} = 400 nm; λ_{EM} = 500 nm; pH = 7.4 (PBS), t = 25 °C}.

Note: the observed decrease of anisotropy can be falsely interpreted as displacement of DIP by the addition of FAVI. In fact, FAVI itself displays fluorescence under these conditions, consequently its contribution to the overall fluorescence anisotropy increases in line with the added FAVI portions. More complicated picture is seen, when the fluorescent competitor binds to the protein as well (and fluorescence intensity of the bound competitor is different from that of the free form), in this case a new component with high anisotropy appears in the system, and the overall anisotropy may even increase in a certain section of the titration curve.

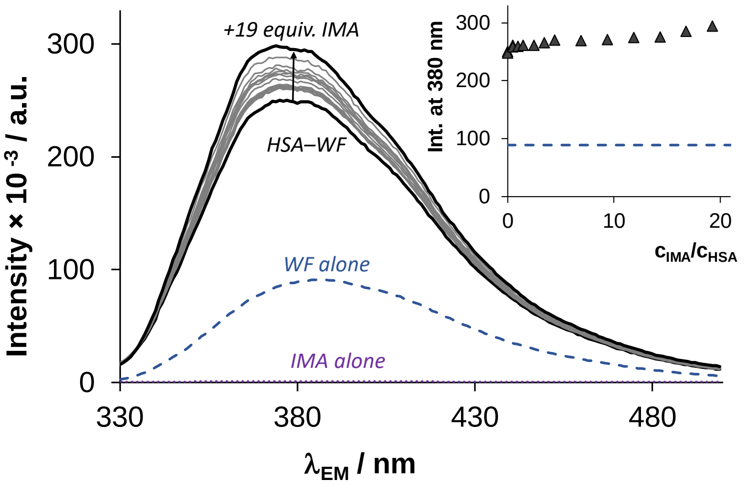

FigureS17. Fluorescence spectra obtained by the titration of HSA–WF (1:1) system with IMA, spectra of WF (1 μM) and IMA (20 μM) are plotted as well for comparison. Inserted figure: emission intensities at 380 nm $\{c_{\mathrm{HSA}} = c_{\mathrm{WF}} = 1.0$ μM; $c_{\mathrm{IMA}} = 0$–19 μM; $\lambda_{\mathrm{EX}} = 310$ nm; pH = 7.4 (PBS); $t = 25$ °C$\}$.

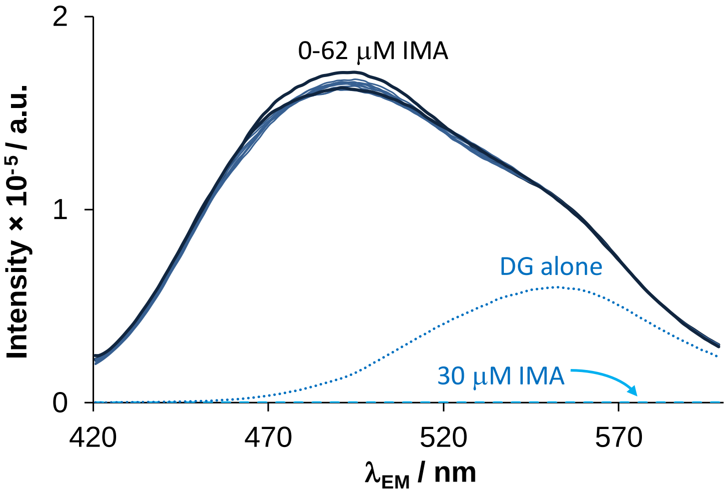

Figure S18. Fluorescence spectra obtained by the titration of HSA–DG (1:1) with IMA $\{c_{\mathrm{HSA}} = 1.0$ μM; $c_{\mathrm{IMA}} = 0$–62 μM; $\lambda_{\mathrm{EX}} = 335$ nm; pH = 7.4 (PBS); $t = 25$ °C$\}$.

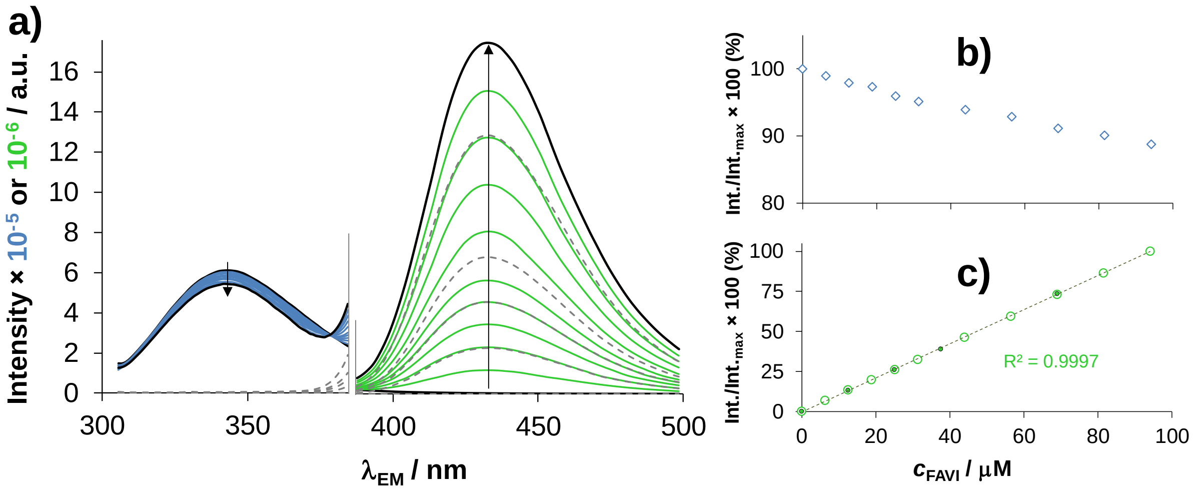

Figure S19. (a) Fluorescence emission spectra of HSA in the presence of FAVI and emission spectra of FAVI alone (green dashed spectra); (b) recorded emission intensities at 340 nm (◊) and (c) 430 nm (○) together with the fluorescence of FAVI alone (●) {c_{HSA} = 2.0 μM; c_{FAVI} = 0–90 μM; λ_{EX} = 295 nm; pH = 7.4 (PBS); t = 25 °C}.

Table S1. Protein bound fractions (%) of IMA, h-MOLNU, MOLNU and FAVI obtained in ultrafiltration experiments at various concentrations {pH = 7.4 (PBS); *t* = 25 °C; equilibration time: 20 min}

		IMA[a] 50 µM	h-MOLNU 30 µM	MOLNU 30 µM	FAVI 25 µM	FAVI 50 µM	FAVI 100 µM	FAVI 200 µM
HSA	630 µM	-	<2	<2	-	-	-	<2
	50 µM	40 ± 10	-	-	-	<2	-	-
AGP	30 µM	-	<2	8 ± 1	<2	<2	<2	<2
	15 µM	-	<2	4 ± 1	-	-	-	<2
blood serum	4-fold diluted	>98	<2	<2[b]	-	18 ± 2	-	-
	2-fold diluted	-	-	-	-	19 ± 2	-	22 ± 2
	non-diluted	-	-	-	-	-	-	28 ± 4
HSA + AGP	630 µM + 15 µM	-	-	-	-	-	-	2 ± 1

[a] Taking into account the considerably high, 85% adhesion to the filter; [b] binding of 50 µM MOLNU: < 2%.

Table S2. Conditions applied for steady-state spectrofluorometric measurements.

Fluorophore	λ_{EX} (nm)	λ_{EM} (nm)	slit widths EX/EM (nm/nm)
HSA (Trp-214)	295	310-450	4/4
AGP	280	290-450	4/4
WF	310	320-500	2.5/2.5
DG	335	420-600	5/5
FAVI	360	370-550	2/2
FAVI (anisotropy)	360	430	4/4
IMA	285	350-550	5/5
DIP (anisotropy)	400	500	4/4
3D spectra	260-400	280-600	depending on the studied system

Table S3. Instrument parameters applied for TCSPC measurements.

Type of experiment	Single λ_{EM}
LED source λ_{EX} (nm)	360
λ_{EM} (nm)	430
Slit width on emission side (nm)	2
Filter type / cutoff (nm) on the emission side	long pass / 370
Count Nr. at peak channel	10 000
Approx. Nr. of channels used for analysis [a]	1400
Time window	100 ns
Time calibration (ns/ch)	0.02532

[a] Total number of channels = 4096.
